# Bladder Cancer in HIV-infected Adults: An Emerging Issue? Case-Reports and Systematic Review

**DOI:** 10.1371/journal.pone.0144237

**Published:** 2015-12-07

**Authors:** Sylvain Chawki, Guillaume Ploussard, Claire Montlahuc, Jérome Verine, Pierre Mongiat-Artus, François Desgrandchamps, Jean-Michel Molina

**Affiliations:** 1 Department of Infectious Diseases, Saint Louis Hospital, Assistance Publique Hôpitaux de Paris, Université de Paris Diderot, Paris 7, Sorbonne Paris Cité, Paris, France; 2 Department of Urology, Saint Louis Hospital, Assistance Publique Hôpitaux de Paris, Université de Paris Diderot, Paris 7, Sorbonne Paris Cité, Paris, France; 3 Department of Biostatistic and Medical Information, Saint Louis Hospital, Assistance Publique Hôpitaux de Paris, Université de Paris Diderot, Paris 7, Sorbonne Paris Cité, Paris, France; 4 Department of Pathology, Saint Louis Hospital, Assistance Publique Hôpitaux de Paris, Université de Paris Diderot, Paris 7, Sorbonne Paris Cité, Paris, France; University of Pittsburgh Center for Vaccine Research, UNITED STATES

## Abstract

**Objectives:**

Non-AIDS-related malignancies now represent a frequent cause of death among HIV-infected patients. Albeit bladder cancer is one of the most common malignancies worldwide, it has been rarely reported among HIV-infected patients. We wished to assess the prevalence and characteristics of bladder cancer in HIV-infected patients.

**Methods:**

We conducted a single center retrospective study from 1998 to 2013 in a university hospital in Paris. Cases of bladder cancer among HIV-infected patients were identified using the electronic records of the hospital database and of the HIV-infected cohort. Patient characteristics and outcomes were retrieved from patients charts. A systematic review of published cases of bladder cancers in patients with HIV-infection was also performed.

**Results:**

During the study period we identified 15 HIV-infected patients (0.2% of the cohort) with a bladder cancer. Patients were mostly men (73%) and smokers (67%), with a median age of 56 years at cancer diagnosis. Bladder cancer was diagnosed a median of 14 years after HIV-infection. Most patients were on ART (86%) with median current and nadir CD4 cell counts of 506 and 195 cells/mm^3^, respectively. Haematuria (73%) was the most frequent presenting symptom and HPV-associated lesions were seen in 6/10 (60%) patients. Histopathology showed transitional cell carcinoma in 80% and a high proportion of tumors with muscle invasion (47%) and high histologic grade (73%). One-year survival rate was 74.6%. The systematic review identified 13 additional cases of urothelial bladder cancers which shared similar features.

**Conclusions:**

Bladder cancers in HIV-infected patients remain rare but may occur in relatively young patients with a low nadir CD4 cell count, have aggressive pathological features and can be fatal.

## Introduction

Due to the long-term efficacy of antiretroviral therapy (ART) and the associated increase in life-expectancy of HIV-infected patients, cancers now represent up to one third of all causes of deaths among HIV-infected patients [1]. AIDS-related malignancies in HIV-infected patients are mainly related to current immunodeficiency and viral infections, but other factors such as a low nadir CD4 count may also be involved [2–3]. Also, non-AIDS-related malignancies are an increasing cause of death, up to 22%, among HIV-infected patients in France [1].

While bladder cancer is one of the most common malignancies worldwide, very few cases of bladder cancer in HIV-infected patients have been reported in the literature [4–15].

Based on the observations of a few consecutive HIV-infected patients with bladder cancer in our department we decided to perform a retrospective study to assess the prevalence of this unusual cancer in this population and its characteristics in the context of HIV-infection.

## Methods

We performed a retrospective single centre study from 01/01/1998 to 12/31/2013 at the Saint-Louis hospital in Paris, a University hospital with a large cohort of HIV-infected patients and a department of Urology with expertise in urogenital cancers.

Cases of bladder cancer among HIV-infected patients were first identified through the screening of the hospital database (programme de médicalisation des systèmes d’information: PMSI) for all in and outpatients who attended the Saint-Louis Hospital during the study period with a diagnosis of HIV and/or bladder cancer [16]. We used the keywords: «HIV» and «bladder cancer» of the international classification of diseases (version 10: ICD10). Also, we screened the electronic records of the HIV-infected cohort followed in the department of infectious diseases using the Nadis° database to identified HIV-infected patients with bladder cancer using the key-word “bladder cancer” [17]. No formal ethics approval was requested for this study as patients records were anonymized and de-identified prior to analysis. Furthermore, patients have signed a consent form when enrolled in the Nadis° database to allow research on their anonymized data.

The study objectives were to assess the prevalence, characteristics and outcome of bladder cancers in HIV-infected patients in our hospital. Patients characteristics and outcome were obtained after careful analysis of patients charts. The following characteristics were collected: age, sex, ethnicity, smoking habit, HIV risk factors, duration of HIV-infection, CDC category, CD4 cell count nadir and CD4 cell count at the time of bladder cancer, antiretroviral therapy (ART), plasma HIV RNA level (below or above 200 cp/mL), Human Papillomavirus (HPV)-associated lesions such as cervical or anal carcinoma and condyloma, presenting symptoms, histologic type, staging using the TNM classification, histology grade, detection of HPV in the tumor, symptoms revealing the bladder cancer, treatment and outcome.

We also performed a systematic review of bladder cancers in patients with HIV-infection, following PRISMA guidance for reporting literature review. We searched for published cases of HIV-infected patients with bladder cancer to identify common characteristics with our patients. We used keywords «"HIV"[Mesh] OR "HIV Infections"[Mesh] OR "AIDS"[Mesh] AND "Urinary Bladder Neoplasms"[Mesh] or " Bladder cancer"[Mesh] » in the PubMed database.

## Statistical Analysis

Quantitative variables were described using median (interquatile range [IQR]) and qualitative variables were described using numbers (percentages). Survival was estimated using Kaplan Meier estimator and presented as estimate and 95% confidence interval (95% CI). Prevalence of bladder cancer in our cohort was compared to that of the French general population using 2008 5-year partial prevalence data [18]. To account for the different age and sex structure between the French population and our cohort, standard morbidity ratio (SMR) and its 95% confidence interval (CI) was computed. All analyses were performed using the R statistical software version 3.0.2. (http://www.R-project.org).

## Results

Among 6353 patients with HIV-infection and 2200 patients with bladder cancer seen at the Saint-Louis hospital from January 1^st^, 1998 to December 31^st^, 2013, we identified 21 HIV-infected patients with bladder cancer using the PMSI database. One additional patient was found using the Nadis° database. We studied all 22 medical charts and seven patients were excluded because they had no cancer (n = 3), were HIV-negative (n = 2), had bilharziosis without cancer (n = 1) or prostate cancer (n = 1).

We eventually included in our analysis 15 patients with a definitive diagnosis of HIV-infection and bladder cancer identified by pathological examination, representing 0.2% of the entire cohort. Five-year partial prevalence (2004–2008) was estimated at 128.1 per 100,000 persons in our cohort. In men partial prevalence was 84.8 per 100,000 men in our cohort as compared to 107.7 in the French general population, and in women 261.4 per 100,000 as compared to 19.7 in the French general population. The global SMR was estimated at 3.44 (95% CI: 0.89–7.65). All patients except one had HIV-infection diagnosed before bladder cancer. This single patient had a diagnosis of bladder cancer made several months before the diagnosis of HIV but at the time HIV-infection was identified, he presented with a very low CD4 cell count suggesting that he had been infected before the diagnosis of bladder cancer.

Data from our patients are summarized in Table 1. Bladder cancer was diagnosed a median of 14 years after HIV-infection in patients with a median age of 56 years (interquartile range: 47–60). Patients were mostly men (73%) and current smokers (67%) with a median of 38 pack-years of cigarette smoking. Their median nadir CD4 cell count was 195 cells/mm^3^, and 55% had a prior diagnosis of AIDS. At the time of bladder cancer, median CD4 cell count was 506 cells/mm^3^, and most patients were receiving antiretroviral therapy (86%), and had suppressed viral replication in plasma (64%). Interestingly, 6 out of 10 assessable patients had HPV-related lesions (cervical cancer in 2, and anal condyloma in 4).

**Table 1:** Patients Characteristics.

No. of patients	15	N missing values
Variables	n (%) or med (IQR)	
**Patients' characteristics**		
Age at bladder cancer diagnosis (years)	56 [47–60]	
Male gender	11 (73)	
White	15 (100)	
Smokers (current)	10 (67)	
**HIV infection**		
Transmission group		
Men who have sex with men	5 (33)	
Heterosexual	2 (13)	
Intravenous drug user	2 (13)	
Transfusion	1 (7)	
Unknown	5 (33)	
Prior AIDS	6 (55)	4
CD4 count nadir (cells/mm^3^)	195 [95–262]	5
CD4 count at bladder cancer diagnosis (cells/mm^3^)	506 [228–703]	6
Plasma HIV RNA <200 at bladder cancer diagnosis (cp/ml)	7 (64)	4
Antiretroviral therapy at bladder cancer diagnosis	12 (86)	1
HPV related lesions		5
Cervical carcinoma	2 (20)	
Anal condyloma	4 (40)	
No	4 (40)	
**Bladder cancer**		
Time between HIV-infection and bladder cancer diagnosis (years)	14 [10–20]	4
Initial symptoms		
Haematuria	11 (73)	
Irritative voiding symptoms	4 (27)	
Histological type		
Transitional Cell Carcinoma	12 (80)	
Epidermoid Carcinoma	2 (13)	
Sarcomatoid Carcinoma	1 (7)	
T stage of the tumor		
a	4 (27)	
1	4 (27)	
2	7 (47)	
High histologic grade	11 (73)	
In situ carcinoma	3 (20)	

IQR: Interquartile Range.

We did not find in our study occupational exposure to chemical carcinogens that could contribute to bladder cancers, there was no case of bilharziosis, and only 1 patient had a familial history of bladder cancer.

Hematuria was the most frequent presenting symptom (73%) and only 27% had irritative voiding symptoms. Cystoscopy was performed in all patients and biopsies allowed the diagnosis of bladder cancer. Histopathology showed urothelial i.e., transitional cell carcinoma (TCC) in 80%, 20% of which had squamous differentiation. High grade histology was found in 11 patients (73%) and 7 patients (47%) had T2 stage tumors with invasion into muscle. In one case HPV was detected by PCR in a tumor biopsy.

Two patients had metastatic disease at diagnosis. Eight patients (53%) underwent transurethral resection of the bladder tumor, three of whom benefited from mitomycin C instillations. Four patients underwent radical cystectomy, two of whom with neoadjuvant chemotherapy, one received chemotherapy and radiation therapy, two only had palliative care due to the tumor extension.

Cancer relapse was seen in 5 patients during follow-up. One-year survival rate was 74.6% (95% confidence interval: 53.3–100). Four patients died during follow-up (27%), all deaths were related to the bladder cancer.

**Fig 1:**
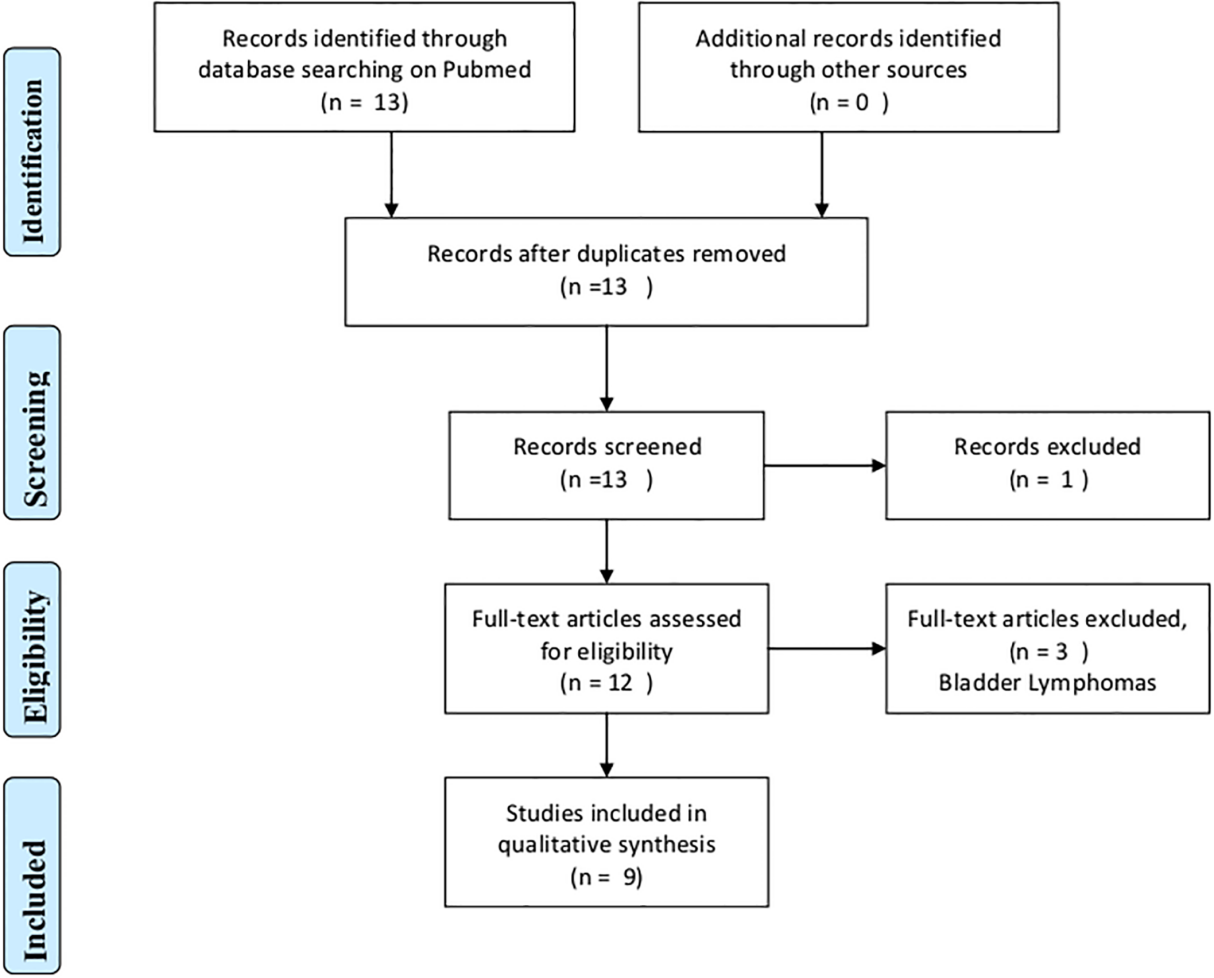
PRISMA Flow Diagram of urothelial bladder cancers in HIV-infected patients.

The systematic review of bladder cancers in patients with HIV-infection identified 13 studies through database searching on Pubmed, and 9 studies were included in the final review (Fig 1) providing detailed information on 16 cases [5–15]. Three cases of lymphoma of the bladder have been reported in severely immunocompromised patients [12, 14–15]. Thirteen cases of urothelial carcinoma were eventually selected for analysis, and Table 2 provides individual data for each case included in this systematic review (S1 Checklist). Most patients were males (11/13), median age at cancer diagnosis was 55 years, and median CD4 cell counts was 299 cells /mm^3^. Eight patients were receiving combined ART and two only AZT. Haematuria was the presenting symptom in 9/12 patients, but two patients with spinal cord injury and neurologic bladder were mildly symptomatic (cases 12 and 13). Histologic type of the bladder cancer was TCC in 11/13 with high grade tumors in 9/11. Death rate was high with 6/13 (46%) dying of bladder cancer during a median follow-up of 8 months.

**Table 2:** Cases of urothelial bladder cancers in HIV-infected patients reported in the Literature.

Case	Ref.	Age (years)	Sex	Follow up	Death from cancer	CD4 (cells/ mm^3^)	Plasma HIV RNA (cp/ml)	ART	Histological type	Stage	Presenting Symptoms
1	11	49	M	1 Year	No	270	ND	AZT	TCC low grade	T1	Dysuria, frequency, lower abdominal pain
2	10	33	M	5 Months	Yes	106	ND	AZT	TCC high grade	ND	Dysuria, Hu, weight loss
3	8	58	M	3 Years	No	572	1600	Yes	TCC low grade	Ta	Hu
4	9	37	F	ND	No	318	ND	No	TCC high grade	T1	Hu, lower abdominal pain
5	13	51	M	4 Months	No	228	ND	ND	SCC	ND	ND
6	8	57	M	8 Months	No	920	< 20	Yes	TCC high grade	T1	Hu (microscopic)
7	5	61	M	7 Months	Yes	228	<50	Yes	TCC high grade	T1	Hu
8	5	49	M	1 Year	No	280	<50	Yes	TCC high grade	Ta	Hu, dysuria, urgency
9	5	63	M	6 Months	Yes	317	50000	Yes	TCC high grade	T3	Hu
10	5	55	M	3 Years	Yes	280	<50	Yes	TCC high grade	T1	Hu
11	5	67	F	ND	No	445	<50	Yes	TCC high grade	T4	Hu, lower abdo pain
12	6	44	M	5 Months	Yes	440	<75	ND	TCC high grade	T4	asymptomatic
13	7	59	M	11 Months	Yes	ND	undetectable	Yes	SCC	T3	Lower abdominal pain

Ref.: references, ND = No Data, ART = antiretroviral therapy, AZT = only treated with zidovudine, TCC = Transitionnal cell carcinoma, SCC = Squamous cell carcinoma, Hu = hematuria, M: male, F: female.

## Discussion

Our study shows that bladder cancer can occur in patients with HIV-infection although it remains a rare event seen in only 0.2% of our cohort in Paris. The five-year partial prevalence of bladder cancer (2004–2008) in our cohort was estimated at 128.1 per 100,000 persons and was similar to that of the French general population in men. When accounting for the different age and sex structure of our cohort and of the French general population however, the global SMR was estimated at 3.44 (95% CI: 0.89–7.65), suggesting that we have observed more cases of bladder cancers than expected. The prevalence of bladder cancer in HIV-infected patients remains however much lower than that of other malignancies in this population, with prevalence rates of Kaposi sarcoma and non-Hodgkin lymphomas reported to be ten-fold higher, and prevalence rates of anal and lung cancers reported to be 5-fold higher on average in 2006 in France [19]. Indeed, bladder cancers have been rarely reported in the literature among HIV-infected patients, with a few case-reports. Recent epidemiologic studies have also reported a low prevalence of bladder cancer in HIV-infected patients, similar or even lower than in the general population, but these studies have been performed in patients with AIDS and might therefore have overlooked the real prevalence of bladder cancer today in the HIV population on ART [20].

Our study and literature review showed that bladder cancers in HIV-infected patients share common characteristics with bladder cancers in non HIV-infected individuals, such as a male sex predominance, higher prevalence in Caucasians, a high proportion of smokers, painless haematuria as the main presenting symptom, and histopathology yielding mainly urothelial (transitional cells) carcinomas which account for 90% of all bladder cancers.

There are however distinct features of bladders cancers in HIV-infected patients that may suggest a potential increase in the incidence of this cancer in the near future in this population.

Indeed, in the general population bladder cancers usually occur at a median age of 72 years in males and 77 years in females, much older that the median age of most cohorts of HIV-infected individuals [4]. Interestingly, the median age of patients with bladder cancer both in our study and in the literature appears to be at least 10 years younger on average than in the general population suggesting that,according to the increasing life-expectancy of HIV-infected patients with ART today, our patients may experience even more bladder cancers in the future. Indeed, the proportion of bladder cancers in a recent study increased by at least 5-fold in HIV-infected patients above 50 years old as compared to those between 40 and 49 [20].

Smoking is a well-known risk factor for bladder cancer and cigarette smoke is responsible for approximately one-half of cases of urothelial cancer in both men and women. The proportion of smokers in HIV-infected patients is usually higher than in the general population putting them therefore at higher risk for bladder cancer. In nationwide study in Denmark, 76% of the HIV-infected patients were current smokers as compared to only 39% of the control population. Smoking-related cancers accounted for 23% of cancers in the HIV-infected population and the risk of these cancers was increased by 2.8 fold as compared to the control cohort [21]. A similar high percentage of smokers was reported in our study (67%). These data altogether suggest that smoking cessation should be emphasized in HIV-infected patients especially as patients are getting older.

The role of immune suppression in the development of AIDS-related and non-AIDS related malignancies has been well established especially for virally-induced cancers such as EBV, HHV-8, HBV/HCV and HPV-related cancers [2–3]. In our study patients had no severe immune deficiency with a median CD4 cell counts above 500 cells/mm^3^, however their median CD4 cell count nadir was low, and a low nadir CD4 cell count has been associated with an increased risk of non-AIDS related cancers such as HPV-related anal cancers [3].

A relationship between HPV infection and urothelial bladder cancer has also been suggested by a number of studies and HPV can infect the epithelia of the bladder. Condyloma acuminatum, characteristic of HPV infection has been reported in the bladder. A recent meta-analysis found that the prevalence of HPV among cases of bladder cancers could be as high as 17% [22]. In our study HPV was clearly detected by PCR in the tumor biopsy in only one case, but a sizable proportion of our patients had HPV-related lesions at the time of cancer diagnosis, suggesting that HPV infection might be associated with bladder cancer especially among HIV-infected patients. More studies should be performed to assess the prevalence of HPV in bladder cancers in the HIV-infected population and its role in the pathogenesis of urothelial cancers.

Interestingly, although the majority of tissue biopsies in our patients showed TCC tumor cells as expected, non TCC tumors were also observed. Also, 20% of TCC tumor had squamous differentiation suggesting a potential role again for HPV infection as a risk factor.

Another unique feature of these bladder cancers in HIV-infected patients was the high rate of aggressive tumors with muscle invasion in 46% and high grade histology in 73%, which can explain the poor outcome of our patients with a case-fatality rate of 27%, much higher than in the general population.

Our study has clear limitations due to its retrospective nature, its limited size, and the lack of systematic search for HPV DNA in tumor biopsies.

Physicians in charge of HIV-infected patients should be aware that their patients, as they become older, face an increased risk of bladder cancer, especially those with a low nadir CD4 cell count, who smoke, and also potentially those with HPV infection. Also, because bladder cancers in HIV-infected patients seem to be more aggressive, this diagnosis should be suspected in any patient with haematuria or dysuria and should prompt rapid urologic examination.

## Supporting Information

S1 Checklist PRISMA 2009 Checklist. (DOC)
